# Effect of mechanical disruption on the effectiveness of three reactors used for dilute acid pretreatment of corn stover Part 2: morphological and structural substrate analysis

**DOI:** 10.1186/1754-6834-7-47

**Published:** 2014-04-01

**Authors:** Peter N Ciesielski, Wei Wang, Xiaowen Chen, Todd B Vinzant, Melvin P Tucker, Stephen R Decker, Michael E Himmel, David K Johnson, Bryon S Donohoe

**Affiliations:** 1 Biosciences Center, National Renewable Energy Laboratory, 15013 Denver West Parkway, Golden, CO, 80401, USA; 2 National Bioenergy Center, National Renewable Energy Laboratory, Golden, CO, USA

**Keywords:** Biomass conversion, Dilute acid pretreatment, Severity factor, Quantitative image analysis, Delamination, Nanofibrillation

## Abstract

**Background:**

Lignocellulosic biomass is a renewable, naturally mass-produced form of stored solar energy. Thermochemical pretreatment processes have been developed to address the challenge of biomass recalcitrance, however the optimization, cost reduction, and scalability of these processes remain as obstacles to the adoption of biofuel production processes at the industrial scale. In this study, we demonstrate that the type of reactor in which pretreatment is carried out can profoundly alter the micro- and nanostructure of the pretreated materials and dramatically affect the subsequent efficiency, and thus cost, of enzymatic conversion of cellulose.

**Results:**

Multi-scale microscopy and quantitative image analysis was used to investigate the impact of different biomass pretreatment reactor configurations on plant cell wall structure. We identify correlations between enzymatic digestibility and geometric descriptors derived from the image data. Corn stover feedstock was pretreated under the same nominal conditions for dilute acid pretreatment (2.0 wt% H_2_SO_4_, 160°C, 5 min) using three representative types of reactors: ZipperClave® (ZC), steam gun (SG), and horizontal screw (HS) reactors. After 96 h of enzymatic digestion, biomass treated in the SG and HS reactors achieved much higher cellulose conversions, 88% and 95%, respectively, compared to the conversion obtained using the ZC reactor (68%). Imaging at the micro- and nanoscales revealed that the superior performance of the SG and HS reactors could be explained by reduced particle size, cellular dislocation, increased surface roughness, delamination, and nanofibrillation generated within the biomass particles during pretreatment.

**Conclusions:**

Increased cellular dislocation, surface roughness, delamination, and nanofibrillation revealed by direct observation of the micro- and nanoscale change in accessibility explains the superior performance of reactors that augment pretreatment with physical energy.

## Background

Lignocellulosic biomass is a renewable, naturally mass-produced form of stored solar energy, and interest in its cost-effective conversion to a liquid fossil fuel alternative has increased steadily over the last decade [1]. Challenges to widespread industrialized biomass conversion processes are presented by the structure and composition of the plant cell wall: this complex, composite material comprises multiple biopolymers that impart structural integrity as well as chemical and biological resistance. This inherent recalcitrance greatly impedes enzymatic digestion of the cellulose in biomass and increases conversion costs [2]. Several different thermochemical pretreatment processes have been developed to address this challenge, however the optimization, cost reduction, and scalability of these processes remain as obstacles to the adoption of biofuel production processes at the industrial scale [3]. The process variables used in current pretreatment technologies include concentration of chemical additives, pH, temperature, and reaction time. The performance and costs of pretreatment are directly related to the choice of pretreatment technology and process conditions as well as the maximum expected sugar yield [4]. Additionally, as we demonstrate in this study, the type of reactor in which pretreatment is carried out can also profoundly alter the micro- and nanostructure of the pretreated materials and dramatically affect the subsequent efficiency, and thus cost, of enzymatic conversion of cellulose.

Here we investigate three reactor configurations that are commonly used for pretreatment of biomass at the National Renewable Energy Laboratory (NREL): the ZipperClave® (ZC), steam gun (SG), and horizontal screw (HS) reactors. A more detailed comparison of these reactors is presented by Wang and colleagues in Part 1 of this study. In brief, all three reactors facilitate high temperature, pressurized, dilute acid catalyzed processes, but employ distinct operational modes: the ZC and SG are batch-type reactors wherein the biomass is pretreated and discharged at the end of pretreatment; the HS reactor is a flow-through process in which acid-impregnated biomass is continuously fed into the reactor. With both the SG and HS reactors, the pressurized biomass is explosively decompressed to ambient conditions at the end of pretreatment, while the ZC releases steam pressure into a heat exchanger gradually through a globe valve.

Corn stover feedstock was pretreated in these three reactors using dilute sulfuric acid as a catalyst. The pretreatments were performed under identical severity according to the traditional metric used to describe dilute acid pretreatment severity (time, temperature, and acid concentration), in this case 5 min at 160°C in 2 wt% H_2_SO_4_. Despite the consistency of the pretreatment severity, significant differences in the digestibility of the cellulose in the pretreated samples were found (see Table 1). We thus sought to identify the critical characteristics of the pretreated materials responsible for the observed differences in digestibility. In the companion study published in this issue, Wang and colleagues report on several chemical and physical properties of biomass substrates that are often altered by pretreatment to improve digestibility. Among these, particle size was a feature that emerged as being significantly different among the samples in this study and correlating strongly with glucan release. This result in particular seeded the interest in an additional in-depth study to investigate evidence of mechanophysical disruption of these samples at multiple scales. In this part of the study, we perform a side-by-side comparison of the effects of pretreatment in these three reactors using a multi-scale microscopy approach that included light and electron imaging modes to investigate the morphology and structure of the biomass substrate at the macro-, micro-, and nanostructural scales. In addition, we report several new image analysis approaches that enable direct quantitative comparison of the degree of mechanophysical disruption among these samples.

**Table 1:** Composition of pretreated samples and percentage of glucan released

**Reactor**	**Composition of pretreated (2% H**_**2**_**SO**_**4**_**, 160°C, 5 min) samples**			**Glucan released (%)**
	**Glucan (%)**	**Xylan (%)**	**Lignin (%)**	
ZC	54.0	8.8	24.2	68.7
SG	60.2	4.8	25.0	88.0
HS	57.4	3.2	24.8	95.2

HS, horizontal screw; SG, steam gun; ZC, ZipperClave®.

## Results and discussion

### Corn stover biomass pretreats differently under the same nominal conditions (2 wt% H_2_SO_4_, 160°C, 5 min) in differently designed pretreatment reactors

The motivation for the rest of the analysis presented here is based on the large range in digestibility of the pretreated corn stover samples from different pretreatment reactors. After 96 h of enzymatic digestion, biomass treated in the SG reactor and HS reactors achieved much higher cellulose conversions (88% and 95%, respectively), compared to 69% for the ZC pretreated material. All of the pretreatments did a reasonable job of depolymerizing and removing xylan and none of the minor differences in the composition of the pretreated samples is enough to explain the difference in digestibility (Table 1).

### At the macro-scale, pretreated biomass particles exhibit color changes, clumping, and particle size variability

**Figure 1:**
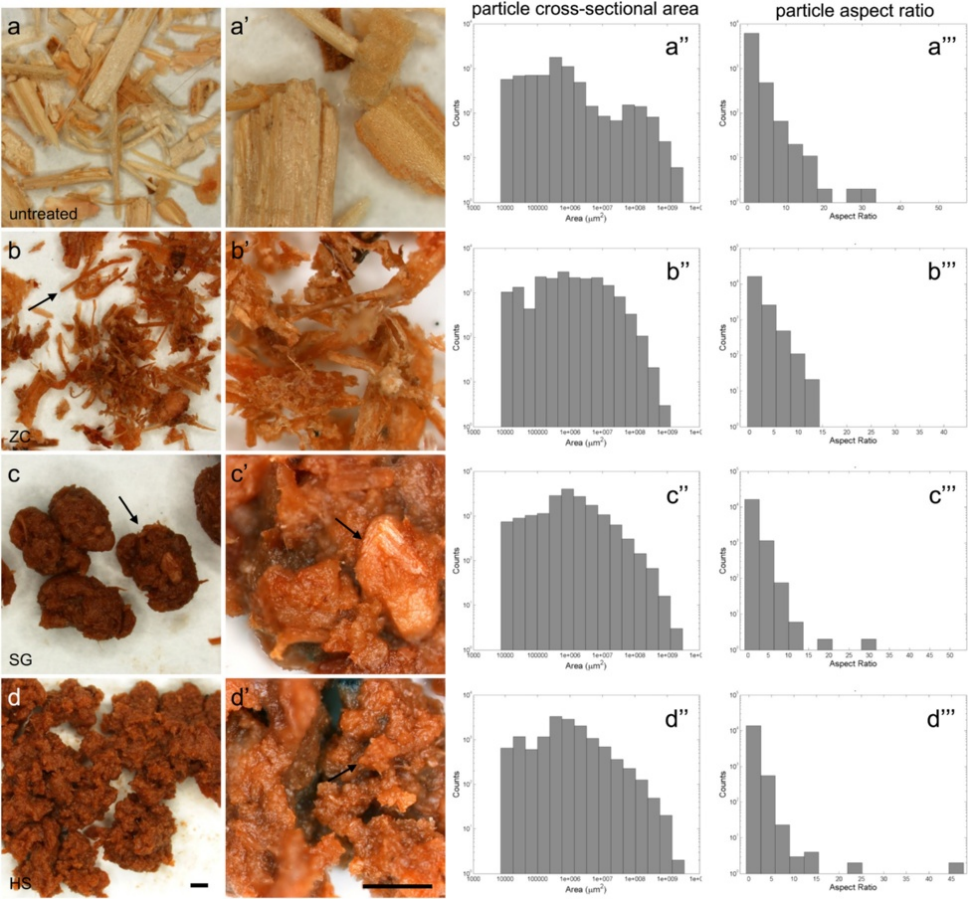
**Stereo micrographs of dilute acid pretreated (2 wt% H**_**2**_**SO**_**4**_**, 160°C, 5 min) corn stover from three different reactors. (a)** Control, **(b)** ZC, **(c)** SG, and **(d)** HS. Samples from all three reactors show a color change to an orange brown color and an observable reduction in particle size. Many long, intact fibers remained in the ZC samples (**b**, arrow). Particles from both the SG and HS reactors clump together (**c**, arrow). The SG caused extensive particle size reduction, but also left a number of larger particles intact (**c’**, arrow). The HS reactor caused extensive particle size reduction to generate uniformly small particles (**d’**, arrow). The shapes of the histograms confirm the observable patterns of reduced particle size of samples from the pretreatment reactors, dramatically reducing the largest (far right) size bins **(a”,b”,c”,d”)**. Also, the reduction in number of long fibers is evident by fewer high aspect ratio counts **(c”’,d”’)**. Bars = 1 mm. HS, horizontal screw; SG, steam gun; ZC, ZipperClave®.

The pretreated corn stover samples from all three reactors were transformed from a blonde tan to an orange-brown color (Figure 1). Even at this scale, morphological differences in the material retrieved from the three different reactors are evident. The ZC left many long, intact fibers (Figure 1b, arrow) and individual particles that do not clump up severely (Figure 1b), whereas particles produced by the SG and HS reactors were extensively clumped (Figure 1c,d). The differences in clumping do not appear to be primarily due to moisture content, because freeze-dried material from the SG and ZC reactors remained as clumped as the wet samples (Additional file 1: Figure S1). In this context, the tendency of particles to clump is more likely attributed to changes in surface morphology, area, and energy; the later effect due, in part, to the re-localization of lignin to the particle surface as reported in previous studies [5,6]. The higher magnification views shown in Figure 1 (a’,b’,c’,d’) suggest that there are differences in the consistency of particle size reduction taking place within the SG relative to the HS reactor. Both sets of samples have undergone size reduction within the pretreatment reactor, but the SG appears to have left more 1 mm or larger sized particles intact (Figure 1c’,d’, arrows).

These data confirm that pretreatment in all three reactors has reduced the overall particle size distribution measured as the area of a two-dimensional projection of the biomass particles and eliminated the bimodal particle size distribution found for the control material (Figure 1a”). Treatment in the SG and HS reactors in particular have shifted the distribution of biomass particles to <1 mm^2^. The observation that the ZC reactor leaves long fibers intact is also confirmed by the particle aspect ratio analysis (Figure 1b”), where more particles with an aspect ratio greater than 10 were found.

By this analysis, the HS reactor succeeded in producing the smallest and most consistently size-reduced particles. Smaller particles, by definition, will have an increased surface area that should promote digestibility. Also, smaller particles with low aspect ratios should have improved mixing behavior. Particles with higher aspect ratios, such as those treated in the ZC reactor, have been shown to impede mixing and reduce the consistency of pretreatment and saccharification within larger batches and at higher solids loadings [7,8].

### Micro-scale evaluation reveals changes in cell wall thickness, cell-cell dislocations, and cell wall fragmentation

**Figure 2:**
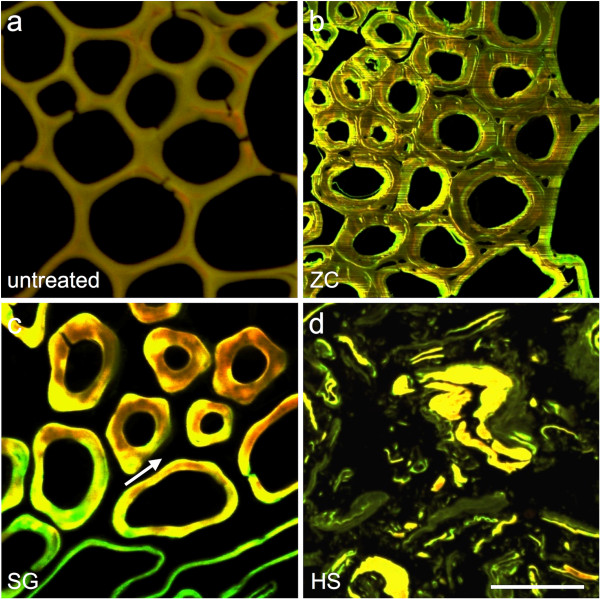
**CSLM micrographs show cellular-scale evidence of biomass deconstruction. (a)** Control, **(b)** ZC, **(c)** SG, and **(d)** HS. The ZC samples have generally thicker cell walls with only limited evidence of delamination and disruptions **(b)**. The SG samples display extensive dislocation of individual cells indicating that the middle lamella layers have been removed and the cells have been forced apart (**c**, arrow). The HS reactor samples also exhibit extensive dislocation, but in addition display cell wall delamination and fragmentation. Bar = 50 μm. CSLM, confocal scanning laser microscopy; HS, horizontal screw; SG, steam gun; ZC, ZipperClave®.

To investigate physical deconstruction phenomena at the cellular-scale, we used confocal scanning laser microscopy (CSLM) of acriflavin-stained sections (Figure 2). Acriflavin is a general purpose stain for lignified secondary walls and its brightness has been reported to be proportional to lignin concentration [9]. The most compelling physical change revealed by CSLM was widespread intercellular dislocation caused by both the SG and HS reactors. Dislocation is evidenced by separation of whole cells from one another at the middle lamella (Figure 2c,d, arrows). This complete removal or relocalization of the middle lamella is known to occur in caustic pretreatments that depolymerize/solubilize lignin and has been described as fiberization in wood pulping processes [10], but this result is not a common feature of dilute acid pretreatments. Neither the control nor the ZC samples showed extensive dislocation; however, the SG and HS reactor samples did exhibit this effect throughout the biomass tissue. Along with dislocation, the HS reactor samples displayed evidence of delamination within cell walls; as well as cell wall fracture and collapse (Figure 2d).

**Figure 3:**
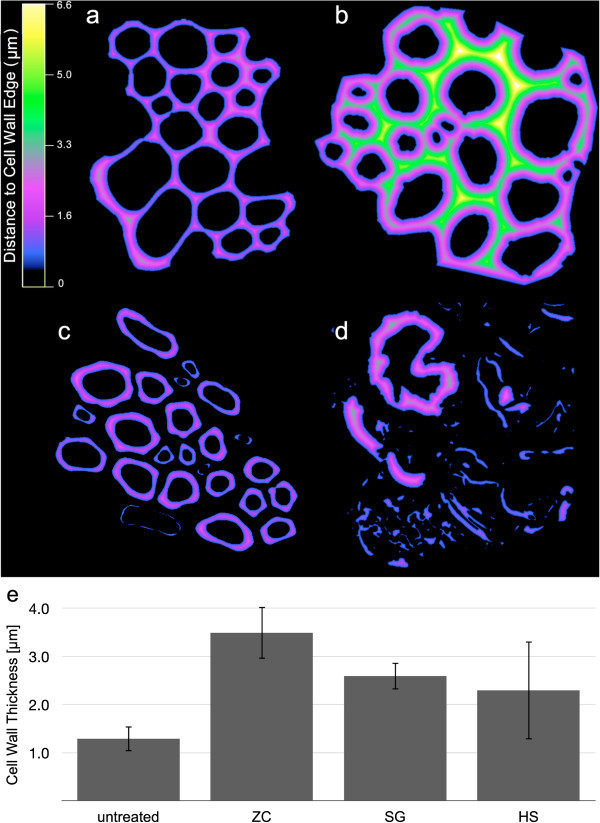
**Distance maps calculated from CSLM images. (a)** Control, **(b)** ZC, **(c)** SG, and **(d)** HS reactor samples pseudo-colored to depict cell wall thickness. **(e)** Mean cell wall thicknesses with standard deviations were determined from the centerline of the cell walls in the distance maps. CSLM, confocal scanning laser microscopy; HS, horizontal screw; SG, steam gun; ZC, ZipperClave®.

Within the vascular bundle fiber cells that were measured, dilute acid pretreatment in each of the reactors caused the walls to swell and increase in thickness. This is consistent with the removal of cross-linking hemicelluloses and the overall impact of pretreatment on increasing the porosity and accessibility of the cell wall to water and enzymes [11,12]. However, increasing cell wall thickness as a result of pretreatment does not correlate well with the increased digestibility, because the SG and HS reactors largely remove the middle lamella from cell walls, producing cell walls that are not as thick as those evident in the less digestible ZC samples. In addition, the HS reactor samples display clear evidence of cell wall fracture and fragmentation at this scale. Those cell wall fragments were included in the cell wall thickness analysis, reducing the average cell wall thickness and contributing to the higher standard deviation in the measurements (Figure 3e). Increasing cell wall thickness is evidence for why the ZC samples are more digestible than the control, but other changes in cell wall structure contribute to the digestibility of the SG and HS samples.

### Surface analysis reveals changes in cell wall roughness due to pretreatment

**Figure 4:**
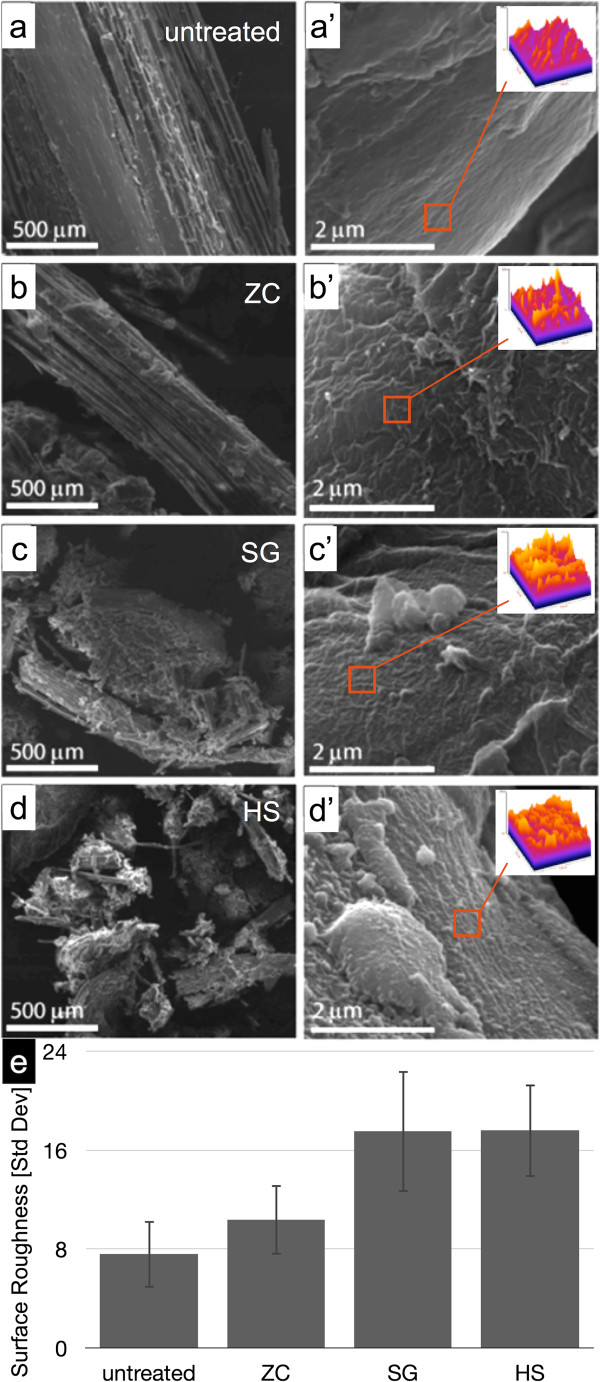
**SEM micrographs of biomass particle and cell wall surfaces. (a,a’)** Control, **(b,b’)** ZC, **(c,c’)** SG, and **(d,d’)** HS. The left column reinforces the particle size reduction and clumping seen at lower resolution in the stereo micrographs. The right column shows changes in surface roughness of the biomass cell walls caused by pretreatment. Surface roughness measurements were calculated as the standard deviation of greyscale values within six selected regions of interest within three different SEM micrographs (orange square, inset). **(e)** The mean standard deviation of the greyscale values and standard deviation among the 18 surface roughness values is reported. HS, horizontal screw; SEM, scanning electron microscopy; SG, steam gun; ZC, ZipperClave®.

Scanning electron microscopy (SEM) analysis was used to evaluate changes in particle deconstruction and particle surface area changes (Figure 4). The left column of Figure 4 (a,b,c,d) shows SEM views of the particle size reduction effect that was also revealed in the stereomicroscope images. The ZC reactor leaves larger, longer fibers intact (Figure 4b) and the HS reactor generates the smallest, most uniformly small particles (Figure 4d). The rightmost column in Figure 4 (a’,b’,c’,d’) shows higher magnification images of the cell wall surfaces that reveal the changes in surface roughness generated in the three reactors. Particle surfaces from all three pretreatment reactors display increased surface roughness compared to the control.

A surface plot visualizing the surface roughness and a chart displaying the averages of the eighteen analyzed regions is shown in Figure 4. The changes in surface roughness measured by this technique trend strongly with the changes in digestibility among the reactor samples and may contribute an important characteristic of effective pretreatment.

### Extensive delamination and increasing intra-cell wall void space is evident at the nanoscale

**Figure 5:**
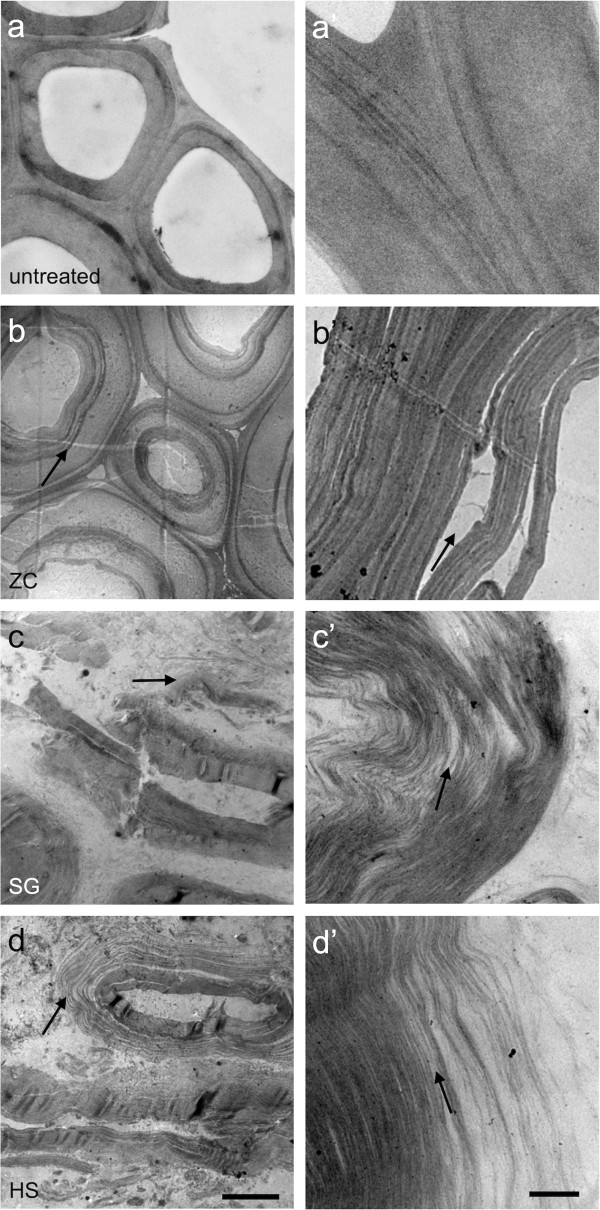
**TEM micrographs display variable degrees of delamination and nanofibrillation within biomass cell walls (arrows). a)** Control, **(b)** ZC, **(c)** SG, and **(d)** HS. The level of delamination and nanofibrillation seen in the SG and HS reactor samples indicate a substantial increase in accessibility **(c’,d’)**. The SG and HS reactor samples also show cell wall fragmentation **(c,d)**. Bars = 5 μm **(a,b,c,d)** and 0.5 μm **(a’,b’,c’,d’)**. HS, horizontal screw; SG, steam gun; TEM, transmission electron microscopy; ZC, ZipperClave®.

**Figure 6:**
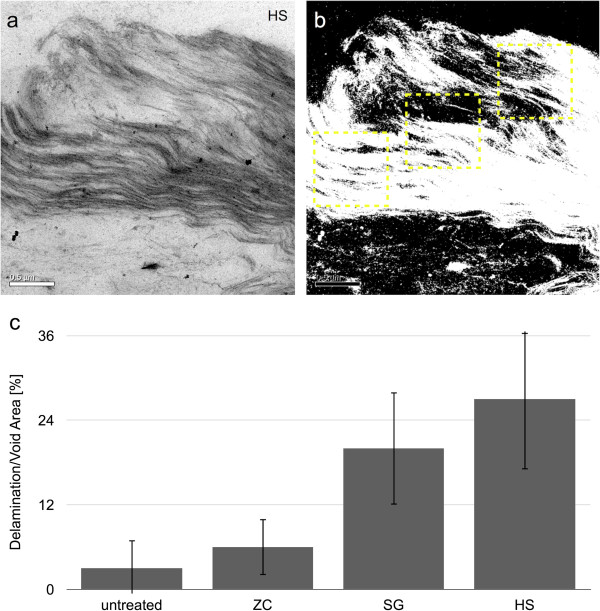
**Determination of delamination and void area from TEM micrographs. (a)** TEM micrograph of a region of cell wall from the HS reactor sample. **(b)** A binary version of the same image that delineates delamination/pore areas and also shows how representative intramural ROIs were chosen. **(c)** Delamination/pore areas measured by image analysis and presented as a percent of the measured area. HS, horizontal screw; ROI, region of interest; SG, steam gun; TEM, transmission electron microscopy; ZC, ZipperClave®.

The differences in cell wall ultrastructure produced by the SG and HS reactor as revealed by transmission electron microscopy (TEM) were striking considering previous imaging analysis on similarly pretreated corn stover samples taken from small-scale batch reactors [5,13]. As reported previously, the ZC samples displayed a characteristic pattern of delamination and relocalization of lignin observed and evidenced by an intramural banding pattern of increasing contrast compared to the control (Figure 5b,b’). We have previously reported some delamination within the S2 layer and between S2 and S3 layers of the secondary cell wall, but the extensively and finely delaminated walls seen in the SG and HS reactor samples is unique (Figure 5c,c’,d,d’, arrows). In addition to the dislocation between adjacent cells and delamination between the major cell wall layers, the SG and HS reactors generated extensive delamination and nanofibrillation throughout the cell wall. Across large areas of the cell wall, it appears that every lamella (that is, a layer of individual cellulose microfibrils and surrounding matrix) has been separated into single microfibrils or small bundles of microfibrils that can be delineated (Figure 5c’,d’). The degree of nanoscale deconstruction displayed in samples treated within the SG and HS reactors can be explained by dramatic increases in the surface area of cellulose microfibrils permitting increased accessibility by hydrolytic enzymes (Figure 6). The quantitative structural parameters obtained by image analysis and several parameters measured in the companion study were correlated to the percentage of glucan released from the biomass from each reactor. These results are summarized in Table 2, and highlight the importance of the morphology of pretreated biomass to its subsequent digestibility.

**Table 2:** Correlations between structural properties and digestibility

**Parameter**	**Glucan release (%)**	**Xylan content (%)**	**Lignin content (%)**	**Degree of polymerization**	**Particle size**	**Aspect ratio**	**Cell wall thickness**	**Surface roughness**	**Delamination/ porosity**
Untreated	24.0	22.0	12.3	2,000	0.15	1.78	1.29	7.60	3.41
ZC	68.7	8.8	24.2	1,750	0.08	1.90	3.49	10.37	5.62
SG	88.0	4.8	25.0	1,650	0.08	1.67	2.60	17.53	20.01
HS	95.2	3.2	24.8	1,850	0.07	1.49	2.30	17.61	26.57
Correlation coefficient (R)		−1.00	0.87	−0.28	−0.97	−0.98	−1.00	0.97	1.00
Correlation coefficient squared (R^2^)		1.00	0.76	0.08	0.94	0.97	1.00	0.94	1.00

HS, horizontal screw; SG, steam gun; ZC, ZipperClave®.

## Methods

Description of the biomass feedstock handling, dilute acid pretreatment in each of the different reactors, compositional analysis, and enzymatic digestibility are described in detail in the companion study, ‘Effect of mechanical disruption on the effectiveness of three reactors used for dilute acid pretreatment of corn stover Part 1: chemical and physical substrate analysis’ in this issue.

### Flatbed scanning

Approximately 100 mg of dried biomass sample was placed inside a plastic envelope and spread to minimize particle overlap or contact. The particles were scanned using an Epson Stylus Photo RX500 (Seiko Epson, Nagano, Japan) flatbed scanner set at 2,400 dpi and captured as TIFF files.

### Stereomicroscopy

Control and pretreated corn stover samples from the three reactors (ZC, SG, and HS) were examined wet and without further processing by stereomicroscopy. A subset of each sample was washed and dried by freeze-drying and imaged dry to reanalyze clumping. Images were captured on a Nikon SMZ1500 stereomicroscope and captured with a Nikon DS-Fi1 CCD camera operated by a Nikon Digital Sight system (Nikon Instruments, Melville, NY, USA).

### Scanning electron microscopy (SEM)

Imaging by SEM was performed using a FEI Quanta 400 FEG instrument (FEI, Hillsboro, OR, USA) under low vacuum (0.40 to 0.65 Torr) operating with the gaseous solid-state detector (GAD) collecting secondary electrons. Samples were washed and prepared for imaging by freeze-drying. Dry samples were mounted on aluminum stubs using carbon tape and sputter coated with 6 nm of gold. Imaging was performed at beam accelerating voltages from 12.5 to 25 keV.

### Sample preparation for microtomy

Pretreated corn stover samples were prepared using microwave processing for electron microscopy as described previously [5]. Briefly, samples were fixed 2 × 6 min (with intermittent power) in 2.5% glutaraldehyde buffered in 1X PBS buffer (EMS, Hatfield, PA, USA) under vacuum. The samples were dehydrated with increasing concentrations of acetone and infiltrated with Eponate 812 resin (EMS) by incubating at room temperature for several hours to overnight in increasing concentrations of resin. The samples were transferred to flat-bottomed capsules and the resin polymerized at 60°C overnight. Embedded samples were sectioned to 300 nm for light microscopy and to approximately 60 nm for TEM with a Diatome diamond knife (Diatome, Hatfield, PA, USA) on a Leica EM UTC ultramicrotome (Leica, Wetzlar, Germany).

### Confocal scanning laser microscopy (CSLM)

Semi-thin (300 nm) sectioned samples were positioned on glass microscope slides and stained with 0.1% acriflavine for confocal scanning laser microscopy of cell walls. Images were captured using a Nikon C1 Plus microscope (Nikon, Tokyo, Japan), equipped with the Nikon C1 confocal system with four lasers (403 nm, 561 nm, 643 nm, and Argon tunable 458/477/488/515 nm), and operated via Nikon’s EZ-C1 software.

### Transmission electron microscopy (TEM)

Ultra-thin sections were positioned on 0.5% formvar coated copper slot grids (SPI Supplies, West Chester, PA, USA). Grids were post-stained for 1 min with 1% aqueous KMnO_4_. Images were captured with a 4 megapixel Gatan UltraScan 1000 camera (Gatan, Pleasanton, CA, USA) on a FEI Tecnai G2 20 Twin 200 kV LaB6 TEM (FEI).

### Macro-scale particle geometry descriptors

Particle size was characterized by image processing to calculate size and shape descriptors including area, perimeter, aspect ratio, and roundness. Similar methods for quantifying the size and shape distribution of biomass particles using image analysis have been reported previously [14]. In this study, images were thresholded to segregate biomass particles from background and the resulting binary file was processed by watershed analysis to disconnect remaining overlapping particles. Then, the Analyze Particles tool in ImageJ was employed to calculate and output the size and shape descriptors of the biomass particles [15]. A custom MATLAB (MathWorks, Natick, MA, USA) script was used to generate the histograms and tables presented in Figure 1 from the text output from ImageJ particle analysis. The numbers of biomass particles analyzed were 3,899 (control), 8,530 (ZC), 9,333 (SG), and 6,769 (HS).

### Cell wall thickness

Cell wall thickness was measured directly from the CLSM images by a procedure adapted from those used previously to measure cell wall thickness in wood [16,17]. The high contrast provided by the acriflavin-stained, 300 nm thick sections, imaged by CSLM (Figure 3a) allowed images of a group of similar cells, such as vascular bundle fiber cells, to be isolated as a region of interest (ROI), thresholded, and converted to binary easily and accurately (Figure 3b). Two operations, performed in tandem on each image, yield an accurate description of cell wall thickness. A boundary-distance function defined as

(1) $$D_{B}\left(x,y\right)=l\sqrt{{\left(x-x_{B}\right)}^{2}+{\left(y-y_{B}\right)}^{2}}$$

gives the distance of each pixel from the nearest boundary of the thresholded region, where *D*_*B*_(*x,y*) is the Euclidean distance between the pixel at coordinates (*x*, *y*) and the nearest boundary pixel at (*x*_*B*_,*y*_*B*_), and *l* is length of one pixel in microns. Representative distance maps calculated from CSLM images of each treatment type as well as native cell walls are presented in Figure 3d. The medial axis transform [18] (also called skeletonization) of the thresholded image gives the set of coordinates, denoted *MAT*(*x,y*), of the mid-points of the thresholded region within the ROI. Evaluating the boundary-distance function at the coordinates given by the medial axis transform as

(2) $$\mathrm{CWT}=D_{B}\left(\mathrm{MAT}\left(x,y\right)\right)$$

yields a measurement of cell wall thickness (denoted as *CWT*) at each pixel in *MAT*(*x,y*). By this method, thousands of cell wall thickness measurements are obtained from each image. A graphical display of the key image processing steps used to obtain these measurements is presented in Additional file 1: Figure S1. In the case of disjoined cells, like those shown in Figure 3c, the measurement represents one half the actual cell wall thickness. For adjoined cells, the distance map value is the cell wall thickness for each individual cell. These image operations were performed with ImageJ, and the numbers of cells and individual measurements of their walls were 13,183 measurements of 54 cells (control), 15,959 measurements of 50 cells (ZC), 16,259 measurements of 51 cells (SG), and 20,179 measurements of 52 cells (HS), respectively.

### SEM roughness factor

A quantitative estimate of variations in surface roughness may be simply calculated as the standard deviation in pixel intensity from selected and normalized regions of interest from SEM micrographs as demonstrated previously by the analysis of surface roughness of paper fibers [19]. While this is not as direct a measurement of surface roughness as that provided by scanning probe techniques, the contrast generated in an SEM micrograph is directly related to changes in slope of the particle surface [20], and thus provides a relative measure of exposed surface area within a sample set. Six 0.5 μm square regions of interest were analyzed from each of three separate SEM micrographs from each sample condition. Pixel values within selected ROIs were re-scaled to maximize the dynamic range of grayscale values from 0 to 255 to normalize ROIs for quantitative comparison.

### Intra-cell wall void space and nanofibrillation

To quantify the void space created within cell walls by relocation of cell wall matrix material and nanofibrillation of cellulose, TEM images were processed to threshold intra-wall void spaces into regions from which size and shape descriptors could be calculated. Six ROIs from six different images of samples from each reactor configuration were analyzed. Thresholds were determined by first calculating the mean and standard deviation of pixel values within a ROI known to contain only void space, such as the cell lumen. The threshold was then calculated as the value at which pixel values above the threshold were two standard deviations from the mean pixel value of the designated void region as

(3) $$T={\bar{x}}_{v}+2\sigma_{v}$$

where *T* is the threshold value, and ${\bar{x}}_{v}$ and *σ*_*v*_ are the mean and standard deviation, respectively, of the pixel values from a known void region. Graphical examples showing example ROIs of intra-wall regions, as well as the binary images determined using this thresholding method, are shown in Figure 6b.

## Conclusions

Increased dislocation, surface roughness, delamination, and nanofibrillation revealed by direct observation of the micro- and nanoscale change in accessibility explains the superior performance of the SG and HS reactors. Reactor designs that augment pretreatment with physical disruption better overcome biomass recalcitrance. We propose that the enhanced disintegration seen in biomass from the SG and HS reactors is primarily due to mechanical work done on the biomass by rapidly expanding fluid. Both the SG and HS reactors employ an explosive discharge of the pretreated solids. In addition, the HS reactor has a continuous screw feed that imparts mixing and mechanical sheer. This mechanophysical energy disrupts cross-linked matrices and, in addition to acid hydrolysis and temperature, results is an unprecedented degree of cell wall nanofibrillation and explains the increased xylan removal.

## Abbreviations

CSLM: Confocal scanning laser microscopy; HS: Horizontal screw; NREL: National Renewable Energy Laboratory; PBS: Phosphate-buffered saline; ROI: Region of interest; SEM: Scanning electron microscopy; SG: Steam gun; TEM: Transmission electron microscopy; ZC: ZipperClave®.

## Competing interests

The authors declare that they have no competing interests.

## Authors’ contributions

WW, XC, and MPT provided the pretreated biomass samples. PNC and BSD performed the microscopy and quantitative image analysis. TBV assisted with the SEM imaging and analysis. WW performed the enzymatic saccharification assay. DKJ designed the reactor comparison project with assistance from SRD and MEH. PNC and BSD wrote the manuscript. All authors read, edited, and approved the final manuscript.

## Supplementary Material

Additional file 1: Table S1 Composition of untreated corn stover samples. **Table S2.** Summary of qualitative imaging observations. **Figure S1.** Stereo micrographs of freeze-dried samples. **Figure S2.** Measurements of cell wall thickness example. Click here for file
