# Supplementary material for: Effect of mechanical disruption on the effectiveness of three reactors used for dilute acid pretreatment of corn stover Part 2: morphological and structural substrate analysis
Source: Biotechnol Biofuels. 2014 Apr 1;7:47. doi: 10.1186/1754-6834-7-47 (PMC4022059; doi:10.1186/1754-6834-7-47)
Supplement: Additional file 1: Table S1 — Composition of untreated corn stover samples. Table S2. Summary of qualitative imaging observations. Figure S1. Stereo micrographs of freeze-dried samples. Figure S2. Measurements of cell wall thickness example. [file 1754-6834-7-47-S1.pdf]

Table S1. Composition of Untreated Corn Stover Samples.

% Composition of Untreated Corn Stover	
Glucan	34.0
Xylan	22.0
Arabinan	3.1
Galactan	1.6
Acetyl	2.9
Lignin	12.3
Sucrose	4.0
Structural Organics	4.5
Non-Structural Organics	1.2
Water Extractable Others	6.1
Ethanol Extractives	2.2
Total Ash	5.8
Total	99.1

Table S2. Summary of Qualitative Imaging Observations.

Summary of Qualitative Imaging Observations

	Tissues			Cells			Cell Walls		
Sample	**color**	**aggregation**	**moisture**	**morphology**	**dislocation**	**lignin**	**thickness**	**delamination**	**fibrillation**
**untreated**	tan	loose	dry	control	none	diffuse	control	none	none
**ZC**	brown	loose	moist	altered	minor	relocalized	swollen	minor	none
**SG**	brown	clumped	wet	collapsed	extensive	relocalized	swollen	extensive	extensive
**HS**	brown	clumped	wet	fractured	extensive	relocalized	fractured	extensive	extensive

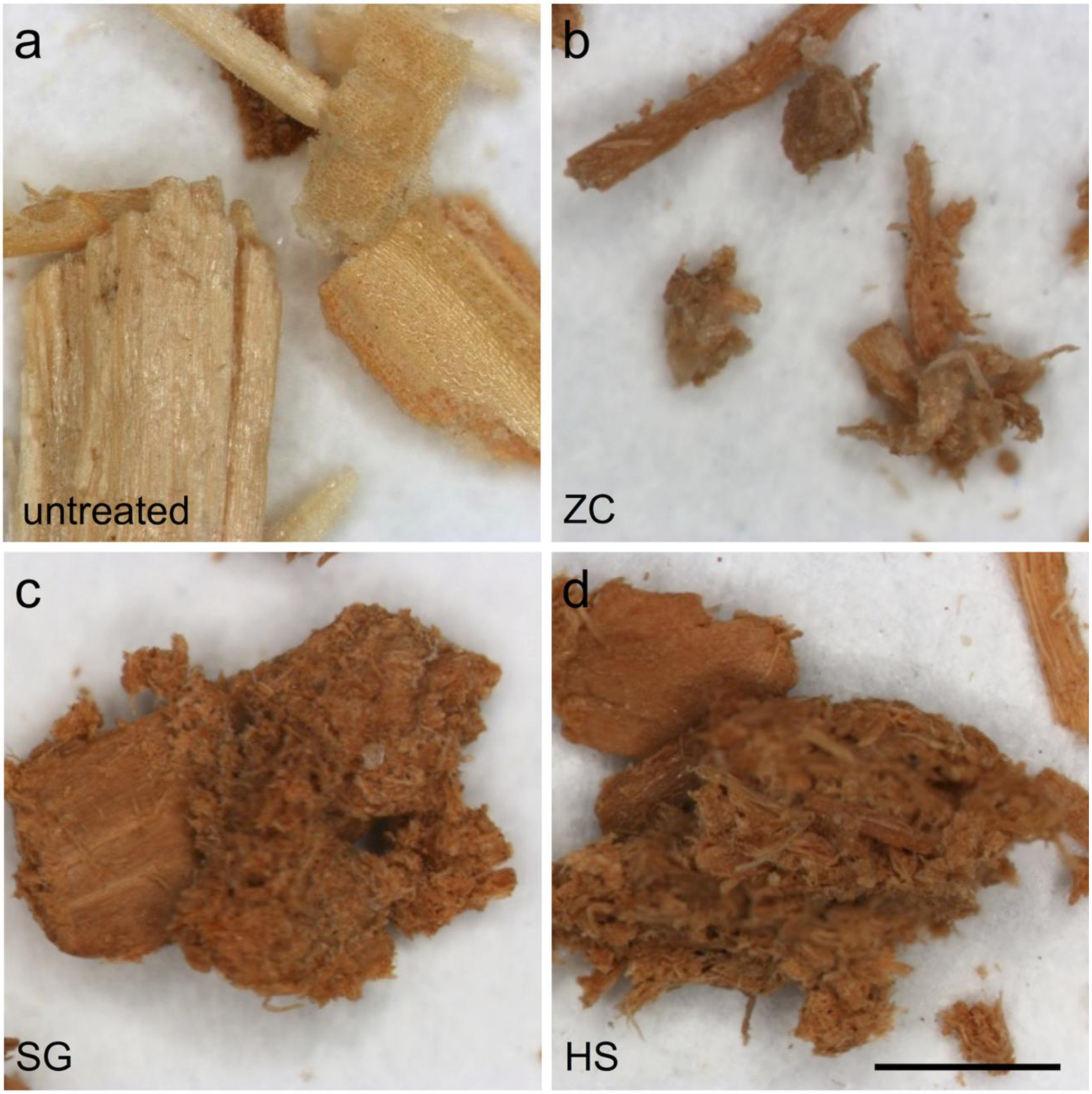

Figure S1. Stereo micrographs of freeze dried samples show that particles from the SG (c) and HS (d) reactors remain clumped even after drying. Bar = 1 mm.

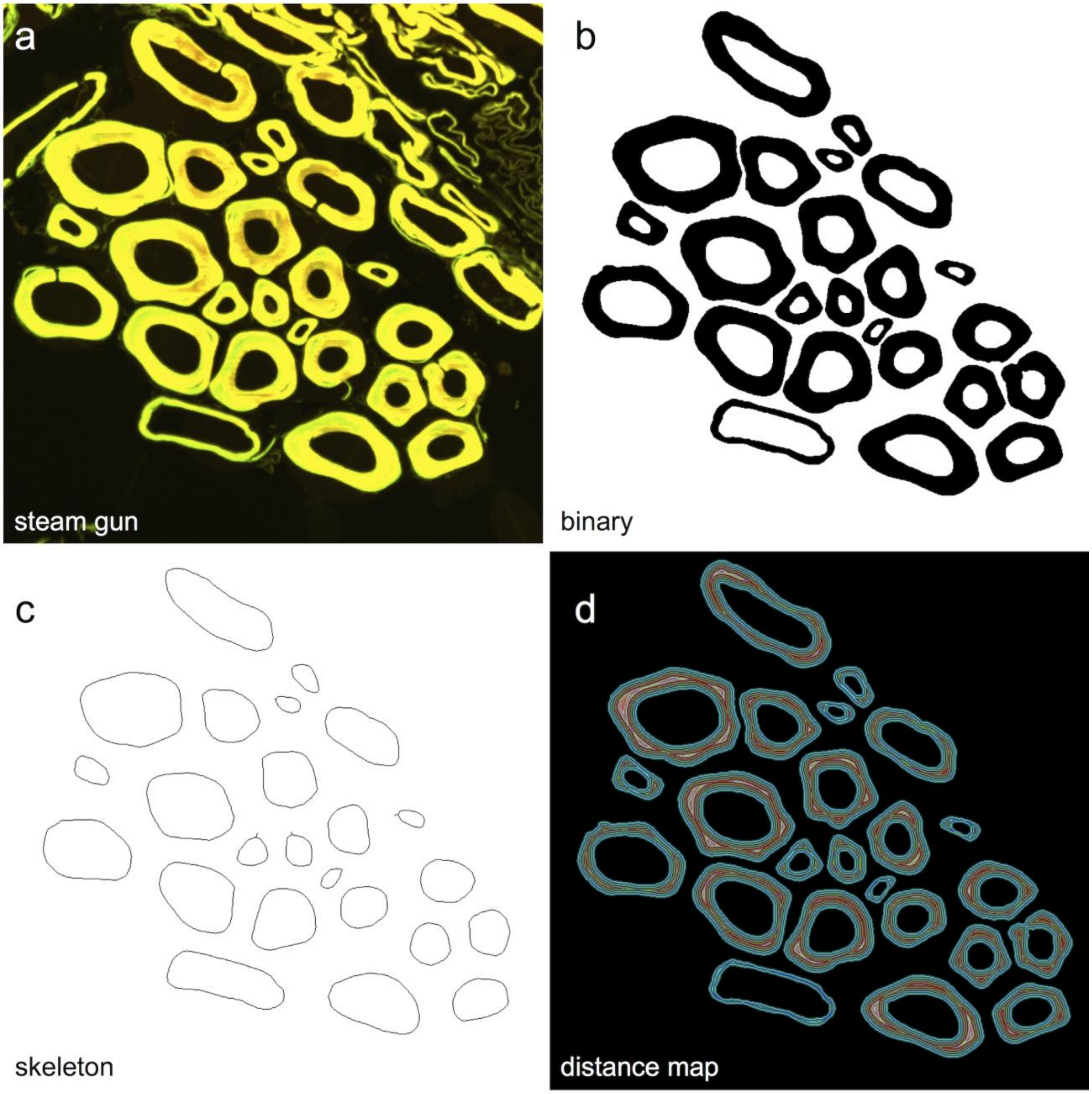

Figure S2. Measurements of cell wall thickness were extracted directly from confocal light microscopy image data (a) by first converting a region of interest within the image to binary format (b). The binary is then converted to a skeleton by a medial axis transform (c) and distance map (d) to derive a distance measurement from the centerline of the cell walls. For connected cells, this measurement reports the distance between the cell lumen and the midpoint of the compound middle lamella. For dislocated cells, it reports one half that distance.
